# The INTERconNEcT-EDs app-based self-help program for people with eating disorders: a usability and qualitative study

**DOI:** 10.3389/fdgth.2025.1629203

**Published:** 2025-11-06

**Authors:** Gaia Albano, Mariarita Semola, Chiara Tosi, Daria Quirino, Elvira Anna Carbone, Dario Gattuso, Cristina Segura-Garcia, Gianluca Lo Coco

**Affiliations:** 1 Department of Psychology, Educational Science and Human Movement, University of Palermo, Palermo, Italy; 2 Department of General Psychology, University of Padua, Padova, Italy; 3 Department of Medical and Surgical Sciences, University “Magna Graecia” of Catanzaro, Catanzaro, Italy; 4 Department of Health Sciences, University “Magna Graecia” of Catanzaro, Catanzaro, Italy; 5 Tech Dgit Easy SRL, Milan, Italy

**Keywords:** eating disorders, guided self-help, usability, digital health, peer support, think aloud tasks

## Abstract

**Introduction:**

Mobile applications for eating disorders (EDs) offer flexible, cost-effective delivery of evidence-based interventions. Nevertheless, challenges persist in terms of user engagement and compliance. The INTERconNEcT-EDs program was developed as a guided self-help (GSH) intervention integrating multimedia content, peer-led support, and group therapy via the aChiral Content app. The present study evaluated the usability and user experience of the aChiral Content mobile application, which was utilized to deliver the INTERconNEcT-EDs program to individuals diagnosed with EDs or disordered eating symptoms.

**Methods:**

A mixed methods design was employed. A total of sixteen participants, comprising eleven outpatients and five members of the community, utilized the application for a period of four days. Quantitative data were collected using the System Usability Scale (SUS), while qualitative feedback was obtained using “think aloud” tasks and a semi-structured interview. The interview-based data was subjected to thematic analysis.

**Results:**

The application attained a mean SUS score of 73.3 (SD = 8.16), denoting satisfactory usability. The analysis of the qualitative feedback indicated that the self-help video-clips and workbook were perceived as being useful, emotionally resonant, and motivating, thus indicating high levels of engagement. The integration of content creators with personal experience of the condition was met with appreciation by users, who characterized this as fostering empathy and perceived support. The forum group and online interpersonal group sessions promoted a sense of community, emotional sharing, and peer support, helping users to feel less isolated. Moreover, certain usability issues were identified and addressed with a view to implementation.

**Conclusions:**

The aChiral Content application exhibited satisfactory levels of usability and acceptability among individuals diagnosed with ED. The integration of user-centred design methodologies, multimedia resources, and the facilitation of peer involvement has been demonstrated to enhance engagement levels. These findings lend support to the potential of the app for wider implementation and scalable use in digital interventions for ED.

## 1 Background

Eating disorders (EDs) represent a significant global health challenge, affecting individuals across diverse demographics and leading to severe physical, psychological, and social consequences (1). The spectrum of EDs, including anorexia nervosa (AN), bulimia nervosa (BN), and binge eating disorder (BED), is characterized by maladaptive eating behaviors, distorted body image, and emotional dysregulation, often resulting in chronic impairment and increased mortality rates (2). Avoidant/restrictive food intake disorder (ARFID) that is characterized by restriction of food intake but does not include having a distorted body image is also a common disorder (3), may have a chronic course that impacts the psychosocial functioning of both the patient and their family (2). Despite the availability of evidence-based treatments, such as cognitive-behavioral therapy (CBT) and interpersonal psychotherapy (IPT), access to care remains limited due to long waiting lists, geographic barriers, and the shortage of specialized clinicians (4). Consequently, there is an urgent need for innovative, accessible, and scalable interventions that can provide timely support to individuals with EDs and prevent the risk of chronicity or symptoms exacerbation.

Digital health technologies have emerged as promising solutions to address the accessibility gap in ED treatment (5–7). There is encouraging evidence that a number of digital interventions for EDs have the potential to deliver evidence-based care in a flexible and cost-effective manner, including the delivery of prevention programmes virtually (8), codesigned serious games as an adjunct treatment and coached CBT (5, 9, 10). Digital interventions can provide real-time symptom monitoring, psychoeducational content, and therapeutic guidance (6, 11) through a personalized support (7, 12). Mobile-device applications can offer additional advantages over computer-based interventions, including their capacity to enabling users to access support at their convenience and time (7, 13). Mobile apps that incorporate features such as peer support, real-time feedback, and therapist-guided interventions have demonstrated higher retention rates and greater improvements in ED-related outcomes (4, 5) and can serve as effective adjunct to clinical care, particularly for individuals in the early stages of ED recovery or those with subthreshold symptoms (14).

Many mobile-based interventions for EDs are developed within the framework of low intensity interventions (15) or guided self-help (GSH), which blends structured self-help materials with minimal professional support (16). This approach has been found to be effective in producing improvements in mild-to-moderate ED symptoms and secondary mental health outcomes, while reducing the burden on healthcare providers (17–19). The inclusion of peer-led support and interactive features further enhances user engagement, as evidenced by the success of apps that integrate social media advocacy and lived-experience content creators (20).

However, there is evidence that adherence to online programs for ED is lower than expected (e.g., completion rates typically below 50%) (6, 21) and new strategies to increase user engagement are crucial (22).

Gathering feedback from users about the characteristics and system function of an app intervention prototype prior to evaluating its efficacy in a large-scale trial can be a user-centered strategy to improve retention and engagement (3, 23). User-centered design and self-reported usability testing are the most used usability approach for digital health apps (3, 24).

Following a user-centered design approach, the current study evaluated the usability of the aChiral app which was developed to host the INTERconNEcT-EDs program, a guided self-help intervention for ED (25). Key features of the app include self-help resources (e.g., workbooks and videos), peer-led interactive support and guided online group sessions targeting psychological distress of people with eating disorders and disordered eating. Both quantitative and qualitative usability metrics will be adopted for the usability test.

## 2 Methods

### 2.1 Study design

A mixed-methods design was employed to evaluate the usability of the aChiral Content mobile application for the GSH program, with a focus on its features and the collection of feedback from individuals with eating disorders. Specifically, both semi-structured interviews and a self-report questionnaire were utilized to gather usability data. The research adhered to the items of the CASP Qualitative checklist. To enhance transparency and rigor in the qualitative component of this study, we applied the Critical Appraisal Skills Programme (CASP) Qualitative Checklist (20). The CASP checklist (26) was utilized as a guiding framework to ensure that the research questions, methodological design, data collection, analysis, and reporting were coherent and adequately justified. The checklist was implemented during the study's design phase and subsequently revisited following the collection and analysis of data, with the objective of ensuring adherence to the qualitative research standards. Furthermore, the reporting of the qualitative component of this study adhered to the 32-item Consolidated Criteria for Reporting Qualitative Research (COREQ) checklist (27). The COREQ framework provides a comprehensive approach to ensuring transparency and rigor in qualitative research reporting across three domains: The three core aspects of the study are as follows: firstly, the composition of the research team and the concept of reflexivity; secondly, the study's design; and thirdly, the analysis of the data and the subsequent findings. In accordance with this framework, the characteristics of the interviewers and their relationship with the participants have been delineated. The study design has been detailed, including recruitment strategies, setting, data collection procedures, and steps taken to reach data saturation. The analytic process has been meticulously delineated, incorporating techniques such as coder triangulation and the utilization of illustrative quotations to substantiate emergent themes. This approach is intended to guarantee that the qualitative findings are presented in a way that is methodologically sound, reproducible, and aligned with international standards for qualitative research reporting (27). The following sections describe the INTERconNEcT-ED(s) program, the aChiral content's features and the data collection processes.

### 2.2 INTERconNEcT-EDs mobile App

#### 2.2.1 Overview

**Figure 1:**
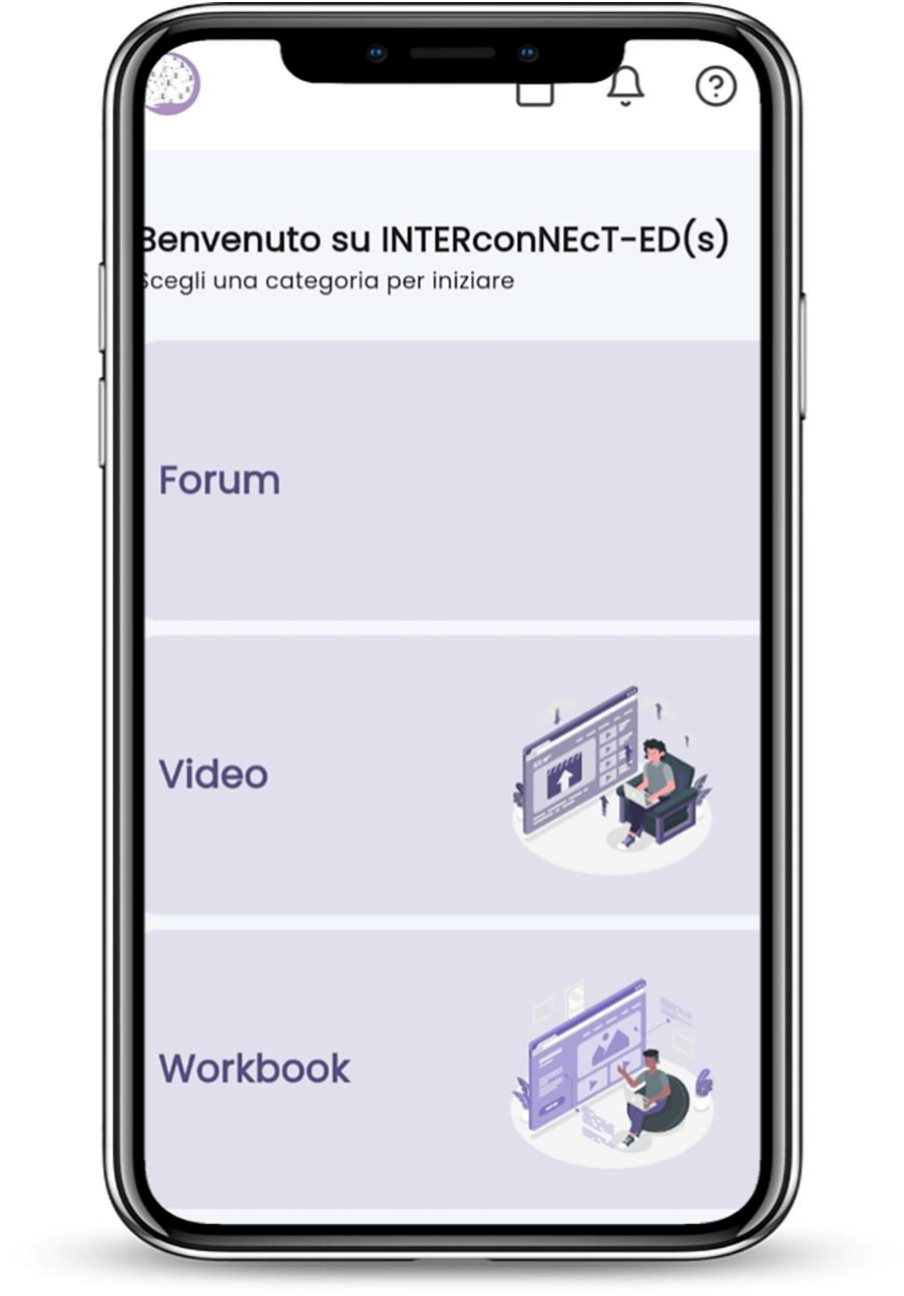
Homepage app aChiral content. Screenshot from: aChiral Content app by Tech dgit Easy.

A prototype of the guided self-help program INTERconNEcT-ED(s) was used and delivered via a mobile app (i.e., aChiral Content) developed by the Tech dgit Easy srl, and available for iOS and Android devices (see Figure 1). The INTERconNEcT-EDs intervention is based on evidence-based, manualized interventions for people suffering from an ED at different stages of the illness (full details of the trial are available at http://www.Clinicaltrials.gov, ID NCT06551974). The aChiral digital health framework integrates aChiral Capture and aChiral Content to streamline clinical research by addressing key aspects of data management and patient engagement. aChiral Capture is a robust Electronic Data Capture (EDC) platform designed to streamline and enhance clinical research data collection while ensuring compliance with FDA (Food and Drug Administration) 21 CFR (Code of Federal Regulations) Part 11 and GDPR (General Data Protection Regulation) regulations. The platform integrates an ePRO (Electronic Patient-Reported Outcomes) module, enabling efficient and secure collection of patient-reported data. For the INTERconNEcT-ED(s) intervention aChiral Capture has been used to address patient engagement, real-time monitoring of patient progress, tailored feedback mechanisms, and data-driven insights to personalize interventions while ensuring compliance with standard care. The framework also incorporates aChiral Capture for EDC (eCRF), enabling efficient and secure data capture of questionnaires and study data, further streamlining clinical research processes.

AChiral Content is a platform that has both a web app and a mobile app. The web app is used to organize the archive of materials, flow of notifications, forum chats and events that the user is going to access from the mobile app during the study. The web app provides functionalities for content and user management, thereby ensuring the consistent delivery of the program through a set of modules, as outlined in Table 1.

**Table 1:** Modules and functionalities of the web app.

Module and functionalities	Description
Personalized Content Feeds	Creation and management of customized content tailored to the individual patient's needs.
Real-Time Updates	Ensure users receive up-to-date content.
Calendar Event Management & Notifications	Reminders for events and groups.
Group Forums and Discussions	A platform for the community to get together and chat.
Feeds Area	Space to see and use materials, forums, events, and exercises, that are organized in categories, with a clear and consistent layout (icons, colours, themes).
Video Content & Workbooks	Access to multimedia content, interactive PDFs, and exercises to complete during the program.
Forum & Group (moderated)	Synchronous and asynchronous discussion spaces on program-specific themes, where moderators make sure that content is appropriate and relevant, and that users don't use unexpected or irrelevant terms and topics.
Bookmarks Library	This section is for the patient to save their favorite content, exercises or feeds from the program. Also, a star rating system lets patients say what they think about the quality of the materials.

These features work together to enhance patient engagement and enrich the overall INTERconNEcT-ED(s) program experience.

**Figure 2:**
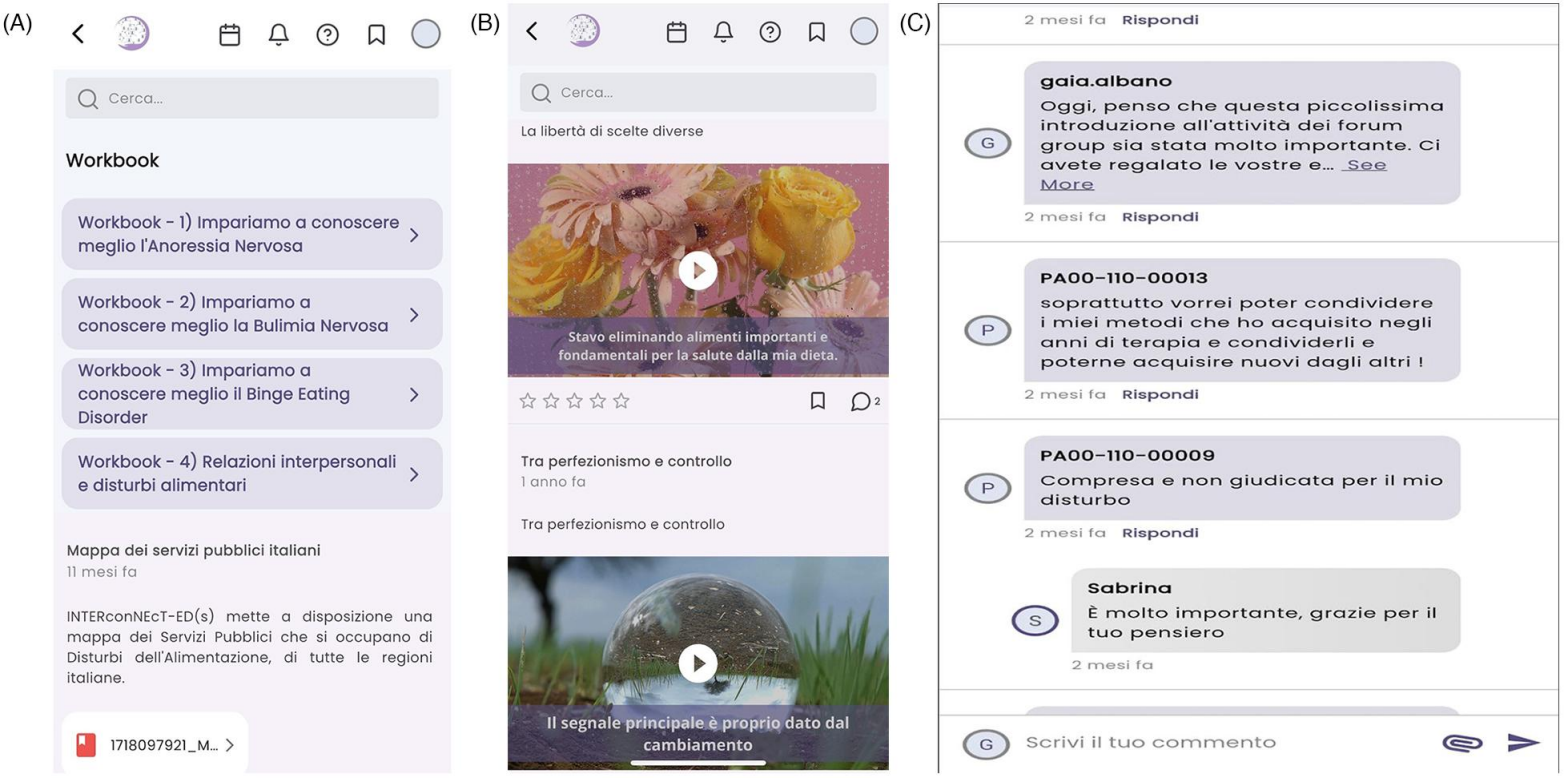
Example of **(A)** workbook, **(B)** video-clips and **(C)** forums group. Screenshots from: aChiral Content app by Tech dgit Easy.

The aChiral Content mobile app was populated with self-help materials developed by professional experts, i.e., psychotherapists and researchers, in accordance with the INTERconNEcT-ED(s) program (25). The selection of written workbooks was made with the objective of providing psychoeducation on eating disorders and interpersonal difficulties, as well as describing and modelling the use of effective strategies to reverse disordered eating behaviors. Moreover, online materials included a library of 74 brief video-clips that were co-designed and recorded with people with lived experience of eating disorders who work as social media content creators, trained and supervised by the research team (see Figure 2). These materials were created through the Instagram profile #DicciComeAiutarti (20).

Participants are also provided with short self-monitoring exercises to facilitate the deepening of their understanding of the content developed and to encourage reflection on their dysfunctional eating behaviors. These exercises are conducted directly through the application via a private comment section, accessible only to the individual user posting the comment and the research team for real-time monitoring.

The transdiagnostic focus of the INTERconNEcT-EDs program on interpersonal relationships is emphasized by an *ad hoc* manual on ED-related interpersonal difficulties, which are considered one of the main factors maintaining the illness (28–30).

A specific section of the aChiral Content mobile app has been designed to host the forum groups. This section contains the weekly, themed, 1 h forum group. The forum groups are synchronous chat sessions and once the activity has finished, mobile app users will have the opportunity to read the full, already closed forum discussion in an asynchronous manner. The contents of the forum groups are moderated by postgraduate students in psychology to prevent the posting of pro-illness contents and the use of a speech that do not respect the personal choices of users. Finally, the INTERconNEcT-ED(s) program offers an additional section for a weekly 90-minute video-conferenced group session (*via* Zoom) led by an experienced therapist using the interpersonal group therapy (IPT) model (31).

### 2.3 Participants

Twenty participants were recruited for the current study. Fifteen outpatients were recruited from the Outpatient Unit for Clinical Research and Treatment of Eating Disorders of the University Hospital “Renato Dulbecco” of Catanzaro (Italy), and five participants were recruited online from the community through the psychoeducation Instagram profile #DicciComeAiutarti (#DCA) (20) which was developed by the University of Palermo. Both outpatients and community people with eating disorders are the target population of the INTERconNEcT-EDs program RCTs (25) and were included in this usability study. The inclusion criteria for outpatients were: (1) age > 18 years, (2) with a clinician-formulated diagnosis of eating disorder based on DSM-5-TR criteria, (3) initiation of outpatient treatment (TAU) within the past four months; (4) access to Internet connection via a mobile device. The exclusion criteria were: (1) a self-reported diagnosis of psychosis, and/or (2) a self-reported neurological impairment. The eligibility criteria for participants recruited from #DCA were: (1) age >18 years, (2) a score > 2.77 on the Eating Disorders Examination Questionnaire (32, 33); (3) not currently receiving a psychological treatment for eating symptoms (i.e., psychotherapy, nutritional and/or psychological counselling, hospital treatment, psychiatric medication for the ED symptoms), (4) access to Internet connection via a mobile device. Prior to participation, all individuals were subjected to a preliminary screening process, which entailed the completion of a concise online checklist that encompassed the specified inclusion and exclusion criteria. After completing the eligibility checklist, participants were personally contacted by the research team by telephone to provide them with the study details, explain the study steps and procedures, and confirm their eligibility and willingness to participate. Four participants discontinued the usability study, and the final sample consisted of 16 participants. The reasons for the dropouts were mainly related to the health status of outpatient participants. In Table 2 are reported the demographic characteristics of the whole sample. Sample size above nine often achieve coding saturation and we expected that the 16 participants enrolled were adequate for the purposes of qualitative analysis (34).

**Table 2:** Characteristics of the sample (*N* = 16).

Variable		Mean (SD) or fr (%)
Age	Outpatients	21,09 (4.5)
	Patients recruited from the community	28,80 (5.404)
Gender	Female	16 (100%)
	Male	0
Recruitment	Outpatients	11 (68,75%)
	Patients recruited from the community	5 (31,25%)
Current treatment	Yes	13 (81,25%)
	No	3 (18,75%)
Eating Disorder diagnosis	Yes	11 (68,75%)
	No	5 (31,25%)
Eds Diagnosis	AN	4 (25%)
	BN	3 (18,75%)
	BED	4 (25%)
	Subthreshold	5 (31,25%)
EDE-Q global	Outpatients	4,51 (1.17)
	Patients recruited from the community	2,41 (1.57)
BMI	Outpatients	26,05 (8.24)
	Patients recruited from the community	28,57 (8.87)

### 2.4 Measures

Participants completed a demographics questionnaire that gathered information about their age, gender, background, and employment status, while additional information on current diagnosis, treatment history, illness duration, and BMI was obtained from clinical records for participants recruited from the outpatient unit, and via self-report for participants recruited from the community.

The Eating Disorder Examination Questionnaire 6.0 (EDE-Q 6.0) (32) was used to assess eating disorder symptoms. It consists of 28 items assessing eating disorder-related thoughts, emotions, and behaviors over the previous 28 days, and a global score is calculated averaging the following subscales: restraint, weight concern, shape concern, and eating concern, with items rated along a 7-point scale (between 0 and 6), where 0 represents “No Days” of eating disorder symptoms and 6 represents symptoms “Everyday”. Higher scores indicate greater eating pathology. In the current study, the EDE-Q showed good internal consistency (*α* = 0.94).

The System Usability Scale (SUS) (35) was used to assess the perceived users' satisfaction and usability of the app. The SUS is a 10-items scale rated on a Likert scale at 5 points (from 5, strongly agree to 1, strongly disagree). Scores from each item are converted to a new number. For odd numbered items, this is the participant's score minus 1. For even numbered items, it is 5 minus the participant's score. These are then summed and multiplied by 2.5 to convert the scores to a scale ranging from 0–100. A score of 68 or more is considered above average usability. In the current study, the SUS showed adequate internal consistency (Cronbach alpha = 0.66).

### 2.5 Procedure

Participants were offered free access to the developed aChiral Content mobile app and its resources for four consecutive days. The four-day exposure period was selected to elicit participants' initial impressions of the app's usability, while also ensuring a reduced participant burden, enhanced retention, and minimized attrition. In view of the objectives of the pilot usability study, priority was given to the provision of rapid and sustainable feedback as opposed to the promotion of long-term application engagement. During these enrolment days, participants had the opportunity to navigate through the mobile app and its services; they were invited to use the self-help resources (workbook and video-clips) and to participate to the introductive forum group facilitated by a selected ED lived experience content creators trained in motivational interviewing and supervised by the research team. Moreover, outpatient participants attended one introductive online interpersonal group session which was led by a group therapist. A welcome mail was sent to all eligible participants, with an introductory video containing key information about participation in the study with the aim to clarify the use of the mobile app and increase participant engagement. Once participants received the first email, they were invited to complete the first task, which consisted of completing the demographic questionnaire via the aChiral Capture platform, and then downloading and accessing the aChiral Content mobile app.

Following the completion of the four-day period of mobile app usage, participants were requested to complete the SUS. Thereafter, they were interviewed by the research team, which comprised four clinical psychologists and researchers (GA, MS, CT and DQ). The team members did not have a clinical relationship with the participants. Specifically, MS and DQ were responsible for patient recruitment from the online community (MS) and the clinic (DQ), respectively. GA and CT conducted the interviews. As illustrated in Table 3, the interview protocol encompassed a range of inquiries concerning the app's usability, along with specific recommendations pertaining to its content and social interaction features. The objective of this approach was to gather comprehensive feedback and proposals for enhancement. The interviews, which ranged from 30–45 min in duration, were conducted via one-to-one videoconferencing sessions involving the participants and a member of the research team. The Google Meet platform was utilized for this purpose, and the sessions were recorded both audio and video. Participants completed the consent form before starting the study. The Ethics Committee of Regione Calabria (Italy) approved the study procedures (ID: 221/2024).

**Table 3:** The interview protocol.

Domain	Example Questions
General Usability	- Do you think the app needs to be changed in any way to make it more useful and helpful for people trying to recover from an eating disorder? - What app features do you not find intuitive? - What do you think about using the app to help with your eating problems? - Would you say that using the app would take up too much time or be too much of a hassle compared to your everyday routine? - On a scale of 1 to 10 (1 = not at all, 10 = definitely), would you recommend INTERconNEcT-ED(s) to a loved one or someone you know who is suffering from an eating disorder?
Self-Help Video Resources	- What did you think about listening to someone talk about what it was like for them to have an eating disorder? - What did you think of the video content? - Were the materials easy to understand? - Did you find the people in the video relatable? - Did the video content match what you were looking for? - Would you suggest that someone with an eating disorder watches these videos?
INTERconNEcT-ED(s) Workbooks	- What did you think of the INTERconNEcT-ED(s) workbook? Did you enjoy the workbook? Were the contents of the workbook easy to understand? - Did you learn something new from this workbook? Did you find the people in the workbook relatable? - Did the workbook cover the things you needed to learn? - Would you suggest that someone with an eating disorder read the workbook?
Group Videoconference Session (only for outpatients)	- Did you like the topics that were talked about in the group session? - What did you think of talking to the other group members on the webcam? - What did you think about the group session? - Would you like to try out an online group programme?
Group Forum	- Did you find the topics interesting and useful? - What did you think of chatting with other users? - What did you think when you read other people's comments on the forum? - Have you learnt anything new from this forum? - Did you find the forum content useful? - Would you tell someone with an eating disorder to join the forum?

#### 2.5.1 Thinking aloud task and semi-structured interview

Usability testing (36, 37) of the aChiral Content mobile app focused on its features and collecting feedback from users at different stages of their ED. The data collection process involved semi-structured interviews with people with EDs, conducted remotely to facilitate direct observation of participant interaction with the platform. Each interview session followed a two-part structure. In the first part, participants were asked to complete the following tasks simulating typical activities: (i) access the aChiral Capture platform (ePRO—Questionnaires), (ii) complete the baseline questionnaires, (iii) access the aChiral Content web platform and reset password, (iv) download the aChiral Content mobile app and log in, (v) viewing and evaluation of self-help video resources, (vi) viewing and evaluation of INTERconNEcT-ED(s) workbook, (vii) access and interaction in group forums, (viii) private commenting on materials, (ix) access to group session via videoconference (only for outpatients). These tasks used the thinking aloud approach (38–40), which encouraged participants to verbalize their thoughts as they navigated the mobile application and platform. This approach provided insights into how participants interacted with the system and identified any usability challenges. Interviewers only intervened when participants struggled to articulate their thoughts, using prompts such as “What are you thinking?” or “What are you trying to do?”. In the second part, participants stopped screen-sharing to answer questions exploring their experiences with the mobile application and its potential integration into their daily lives.

In the same week after three or four days, participants were interviewed by a member of the research team about the usability of the mobile app, the self-help content and their own user experience. The interview and included some additional questions about the participants' overall experience with the program, their motivation for completing the program and whether the user would recommend the INTERconNEcT-EDs program to others. In the course of the semi-structured interview, participants were invited to offer suggestions for the enhancement of the mobile application. The research team then implemented changes to the majority of the items deemed helpful and consistent with the rationale of the intervention (see Table 4).

**Table 4:** Improvements suggested and developed on the mobile app.

Improvements suggested	Improvements developed on the mobile app
Optimize access links to facilitate first login directly into the mobile app	√
Make workbook PDF editable with the ability to underline, add notes on text, add private comment to perform tasks on text via pop-up	√
Optimization of reading workbooks from mobile through screen zoom and rotation	√
Improve the understanding of the user guide, specifying all the application's functionalities and the system of notifications	√
Creation of a section that consolidates all user bookmarks from reading workbooks, enabling quick and easy access to the most frequently used and appreciated sections.	√
Optimize reading messages and moderating the group forum	√
Add a one-to-one chat with the research team	√
Add a personal photo food diary	X
Delete notifications of a new message to the group forum and add reply notifications to single message and message moderation notifications	√
Categorize videos based on the severity of content	X
Improve video descriptions to facilitate the search for videos of interest	√
Click on the “search” button to speed up the search for a topic of interest	√
Reduce workbook length	X
Allow eye contact/chat with peer mentors	X

√, implementable; X, not implementable.

### 2.6 Data analysis

Descriptive statistics, including means and standard deviations, were computed for quantitative data derived from the EDE-Q and SUS by using SPSS (Version 29). Inferential statistics were not used given the small sample size. A deductive thematic analysis (41–43) was used to organize the data using a coding template analysis approach according to the in-depth study of different domains of interest for the effectiveness of mobile apps for mental health (2, 12, 44): (1) usability of mobile app, (2) impact of developed content [INTERconNEcT-ED(s) program], (3) user experience. The deductive thematic analysis methodology involved coding and organizing the data into themes directly related to the features of the mobile app, by allowing for a targeted assessment of usability issues. The content themes were discussed *a priori* by the interviewers and a thematic framework was created for the content developed (video, workbook and interaction material) and the users' experience of interactional guidance (forum group and group session). Investigator and methodological triangulation were adopted to minimize individual biases and ensure a more objective interpretation of the data. The anonymized transcripts of interviews were combined with data collected with the thinking aloud approach, and were coded manually by four independent judges (GA, MS, DQ, CT) without using any software program for the analysis. Manually coding helped judges immerse themselves in the data, leading to a deeper, more interpretive engagement with the material. The coders familiarized themselves with the data through repeated readings and independently identified the initial codes (i.e., coding sentence by sentence). After all transcripts were coded, they were reviewed, and emergent codes were synthesized into subthemes. Coding disparities were identified and discussed as a team until a consensus was reached. Subsequently, the coders worked jointly to review the subthemes for coherence and distinctiveness until consensus among all investigators was reached. Finally, the coded data were reviewed to ensure that the predefined themes accurately reflect the information gathered.

## 3 Results

### 3.1 Quantitative analysis

SUS total and item scores are presented in Table 5. The mean total score was 73,33 (8.16), which is above the cut-off for an acceptable usability. Only 3 participants reported a total SUS score below 68. No missing data were present for the questionnaires.

**Table 5:** Descriptive statistics derived from systems usability scale items (*N* = 16).

Variable (SUS items)	M (SD)	Range
1. I think I would like to use the app frequently	3,00 (0.65)	2–4
2. I found the app to be unnecessarily complex	2,93 (0.88)	1–4
3. I thought the app was easy to use	3,27 (0.59)	2–4
4. I think that I would need support of a technical person to be able to use the app	2,73 (0.80)	2–4
5. I found the various functions in the app were well integrated	2,93 (0.46)	2–4
6. I thought there was too much inconsistency in the app	2,87 (0.74)	1–4
7. I would imagine that most people would learn to use the app very quickly	3,33 (0.49)	3–4
8. I found the app very cumbersome to use	2,40 (0.91)	0–3
9. I felt very confident using the app	3,40 (0.51)	3–4
10. I needed to learn a lot of things before I could get going with the app	2,47 (0.99)	0–4
SUS TOTAL SCORE	73,33 (8.16)	60,00–82,50

### 3.2 Qualitative analysis

The results of the qualitative analysis are presented and discussed in two sections. The first section shows the results of the usability testing, by illustrating the problems identified by the users and linking them to the specific functionalities of the aChiral Content mobile app. The second section explores the users' experiences with the aChral Content mobile app.

#### 3.2.1 Usability data

The user feedback collected through the thinking aloud method is presented in Table 6. It summarizes the users' usability ratings for each platform feature, expressed as tasks in the rows. The columns of the table show, for each feature, both positive and negative evaluations of the functionality, along with any suggestions for improvement to be implemented in development.

**Table 6:** Usability data: users' feedback collected with the thinking aloud.

Functionality (tasks)	Positive evaluations	Negative evaluations	Suggestions for improvements
Get access to the aChiral capture platform (ePRO)	Access considered easy, intuitive, and not onerousness	Entering the One-Time Password (OTP)	N/A
Fill in the requested set of questionnaires and Colour chart	Questionnaires that are easy and quick to complete The section that marks uncompleted questionnaires in red and completed ones in green is intuitive.	Baseline too long Preference for Likert scale questionnaires	N/A
Access to INTERconNEcT-EDs app	The option “save the password” after the first login and access the mobile app via Face ID or Touch ID.	Inserting the access link on the aChiral Content mobile app to make the first login to the mobile app.	Optimize the access link to facilitate the first login from the mobile app.
Viewing and evaluating Self-Help video resources	Immediate availability of Self-Help resources on the home of the aChiral Content mobile app Nice, clear videos, well-organised into different categories It is clear that these are audio interviews: this makes the videos more realistic and easier to relate to Ease of reviewing something interesting The subtitles in the videos are useful	In some videos, the audio is low The star system is confusing with the option to add the video to favourites	Improve the user guide comprehension: specify how to use the star system, clarify that comments on self-help resources are private Add a “search” button to speed up the search for a topic of interest
Viewing and evaluation of the INTERconNEcT-ED(s) Workbook	Immediate availability of Self-Help resources on the home of the aChiral Content mobile app Ease of re-reading something interesting The reading is easy The bookmark is useful for retrieving the reading point	The workbook is too long The star system is confusing with the option to add the video to favourites The use of the bookmark was not clear	Optimise workbook reading on mobile (zoom, screen rotation) Improve the user guide comprehension: specify how to use the star system, clarify that comments on self-help resources are private and specify how to use the bookmark Add a “search” button to speed up the search for a topic of interest
Access and interaction to interpersonal group sessions	The access to the active forum group is intuitive It is useful to be able to reply to a specific message It is useful to have the forum date in the mobile app's calendar	It is difficult to follow the flow of the conversation during the forum group Too many notifications at once for each comment	Optimise message reading and forum group moderation Remove notifications for new comments in the forum and add notifications for replies to individual comments
Homework completion	Completing the exercises directly on the mobile app is considered as a logbook.	It is onerousness and not very intuitive to complete the homework in a section of comments outside the workbook Greater ease in completing the homework on the computer rather than on the smartphone app	Make the workbook PDF editable, with the ability to underline, add notes to the text, and add private comments for activities on the text via pop-up.

#### 3.2.2 Users' experience of navigating the mobile app

The interview-based data were organized into two themes directly related to the features of the mobile app, providing insight into the users' experience of navigating the mobile app aChiral Content: (i) the impact of the INTERconNEcT-ED(s) program and the developed content (i.e., video, workbook and interaction material) and (ii) the role of interpersonal interactions with peer mentors and therapists.

The content developed consists of self-help resources (i.e., videos and workbooks) that users can choose to use in patient-centred self-care. Participants' feedback converged on the following sub-themes related to these contents: user engagement, perceived support, innovation, utility, impact on EDs and clarity.

The aChiral Content mobile app allows users to view materials organized by categories of meaning and content. This feature was found to be intuitive and useful in facilitating their visualization. In particular, the content was rated very positively in terms of:

User engagement (i):

(illustrative quotes)…

“Your voice was really moving. It was very educational because every time I heard a word I kept thinking, ‘Yes, that's true, you're absolutely right, yes, that’s true, you're absolutely right’. .. So, the videos felt like an extra motivation, an extra push in the whole process that I'm going through, and it was really, really important. It was very educational because every time I heard a word I kept thinking, “Yes, that’s true, you're absolutely right, yes, that’s true, you're absolutely right”. “I think there was an opportunity to open up, to feel understood, so you feel like you are in a safe space”. (Participant 1, Female, 19).

Perceived support (ii):

“Especially in the first two videos, I recognized the voices of some girls who share content on social media, and that was wonderful because it means you’ve included the whole area of social media advocacy. This is important because young people in particular are very engaged with these platforms. Even though I feel super old, it’s important to get them interested in these issues, so you did well to include people who are talking about these issues on these platforms”. (Participant 3, Female, 26)

Innovation (iii):

“It was very interesting to be able to interact through messages. For me, writing is very helpful; it’s a way I can express myself more easily, so it was even easier to interact this way”; “The doctors aren’t always available, of course, they can’t keep up because there are other patients as well, so maybe when I go through moments of crises, reading the content or watching a video helps me to feel understood.” (Participant 2, Female, 23)

Utility (iv):

“I was really interested in interpersonal relationships because it’s a big struggle of mine, a hurdle I can never get over, both in my relationships with others and in my relationship with myself. I found it very useful, especially for evaluating how I relate to others, so my relationships, how much importance I give to them. I really liked the exercise on how I relate to others, how I feel. The concept of dominance really made me understand how I position myself and how I always feel inferior to others when in reality we’re all on the same level”. (Participant 5, Female, 23)

EDs impact (v):

“The video about the mountain, where sometimes you have to say “I don't know”, because it’s about letting go of control. I find that very useful, for example, because I'm a very controlling person, so sometimes it feels nice to say ‘I don't know’. It means you don't have to know exactly what to expect, and that’s a bit of a protective factor. Instead of always feeling like I have to know everything and be in control, it’s a lifesaver for me. A safe haven: “Knowing that when I'm having a binge I can write about it and get just one comment from someone who completely understands what I'm going through is essential - I feel understood. Being able to have ongoing sessions with an expert can create a healthy habit of talking about the disorder”. (Participant 3, Female, 25)

Clarity of the proposed content (vi):

- “I found it interesting in relation to my current condition, but if I had been in a more acute phase, I’m not sure how helpful it would have been, as I found some of the content a bit annoying at times.”

or

- “It’s written in a fairly formal way. You can tell that it was written by professionals, but I don't think the terminology is suitable for everyone. Especially for younger people, it’s written in somewhat difficult terms. There’s also a lot of material“. (Participant 14, Female, 26)

In terms of users' experiences of participating in the forum group and the interpersonal group session, the data showed the importance of sharing experiences and interacting with other users who had left similar conditions or with peer mentors; on the other hand, participation and interaction in the interpersonal group session was also considered important and insightful. Comments about the lived experience during the four days of navigating the app were largely similar across participants. The majority of participants reported that the lived experience in the forum group and the interpersonal group session contributed to an increase in positive effects through:

Peer to peer social interactions (i),

(excerpted example)…

“I think it is helpful to connect with other people who have experienced or are going through the same situation. I think there was an opportunity to open up, to feel understood and acknowledged, which gives you the feeling of a safe space”; “For example, the first girl spoke and said that she has been in therapy for a while, that she has moments of anxiety and depression. I saw myself in her and thought: after all, it’s not so bad to talk. She helped me, so I realized I could help others. All you need is a little courage. One thing I noticed was that I didn't feel alone. I could relate to almost all the experiences of other people and that helped me—it means that I can also share my experiences and be of help. I think it’s a nice thing to support each other—Taking part in an online group journey is useful and interesting”. (Participant 13, Female, 32)

Emotional tone (ii):

“The interaction was meaningful and created a supportive environment that made me feel safe and free from judgement. I was often moved, and my sense of loneliness felt a little lighter. Learning about other people’s experiences always gives you something new to compare with what you are going through.”; “It’s nice to connect with other people who, like me, are experiencing this discomfort. It was also valuable to reflect together with the therapist and the other participants on what makes me feel uncomfortable around others who share this struggle or with my family. Listening was really meaningful. It was a pleasant experience to share my thoughts, especially because there was no judgment—only a sense of acceptance. My overall impression was positive, and I was moved in a good way. Hearing about other people’s experiences touched me deeply”. (Participant 11, Female, 22)

Personal Involvement (iii)

“It is convenient because you can see all the messages from the participants. So, even though I might feel embarrassed to write what I think at first, when I see others sharing, I realize that if everyone is participating, why shouldn’t I? The forum gives me a sense of security because as I read other people’s messages, they read mine. But it still feels like a shared conversation where we’re all talking about the same thing and supporting each other, which I think makes it a bit easier. I saw how everyone was trying to help each other. I think the support you feel in the videoconference is also there in the chat between users. I found that people were very similar to each other and to me, so I didn’t feel uncomfortable opening up”. (Participant 5, Female, 19)

Interpersonal sharing (iv):

“You connect with other people—maybe those who have similar struggles. We don’t all share the same experiences, but it makes you feel less alone. Everyone shared their own experiences and it was really nice. The chat is anonymous, so there was no fear of being judged. Personally, I don’t mind, but for others it might be important for privacy and to feel respected. After all, we’re not all at the same stage of our journey”. (Participant 2, Female, 23)

It is worth noting that some participants reported some negative feedback regarding the interpersonal group sessions, mainly due to the videoconferencing format:

“The only thing that’s a bit ‘embarrassing’ is having to turn on the microphone or the camera. I feel a bit awkward because I don’t know the other people. I feel more self-conscious about showing myself to others, even though I believe that sharing my story—as others have done—is mutually beneficial. So, if I have to force myself to do it, I do it. It’s always a bit embarrassing because you never know who’s on the other side. That day, only three of us had the camera on, the others didn’t, so you know there are a lot of people listening to you, but you can’t see them and you don’t know. So, it’s interesting, yes, but I don’t know how much I would like it personally because I’m not very used to it. I’ve always done things alone, so this is a completely new reality.” (Participant 3, Female, 25)

A comprehensive overview of the themes, sub-themes, and feedback received from the semi-structured interviews and think aloud protocol is presented in Table 7.

**Table 7:** Comprehensive overview of the themes, subthemes, and feedback received from the semi-structured interviews and the thinking aloud protocol.

Themes	Tasks	Sub themes					
		*Easiness*		*Intuitiveness*		*Onerousness*	*Utility*
Theme 1: Usability assessment	*Get access to the aChiral capture platform*	+		+		+	n/a
	*Fill in the requested set of questionnaires*	+		+		+	+
	*Download INTERconNEcT-EDs app*	+		n/a		n/a	n/a
	*Access to INTERconNEcT-EDs app*	+		+		-	n/a
	*Viewing and evaluating Self-Help video resources*	+		+		+	+/-
	*Viewing and evaluation of the INTERconNEcT-ED(s) Workbook*	+		n/a		+	+
	*Access and interaction to interpersonal group sessions*	+		n/a		n/a	n/a
	*Homework completion*	+		-		-	+
	Contents	Sub themes					
		*Users Engagement*	*Utility*	*Clarity*	*Perceived Support*	*Innovation*	*Eds Impact*
Theme 2: Content assessment	*Workbook*	+	+	+	+	n/a	+
	*Videoclips*	+	+	n/a	+	+	+
	*Forum groups (Interactional Materials)*	+	+	n/a	+	n/a	+
	Experience	Sub-themes					
		*Peer interaction*	*Emotional tone*		*Involvement*	*Appreciation of delivery modalities*	
Theme 3: Users' experience with interactional guidance	*Forum groups*	+	+		+		+
	*Interpersonal group session*	+	+		-		n/a

“+”: positive feedback; “-”: negative feedback; “+/-”: neutral feedback; “n/a”: absence of feedback.

## 4 Discussion

The current study described the development and usability testing of the aChiral Content mobile app and the aChiral Capture platform for guided self-help in eating disorders [the INTERconNEcT-ED(s) program]. Sixteen participants with an ED or symptoms of disordered eating provided feedback on the usability and acceptability of the mobile app. The results showed a good usability of the aChiral Content mobile app among participants and these findings further support the importance of user-centred design in the development of digital interventions for eating disorders (4).

The mean SUS scores (mean = 73, 33, SD = 8.16) indicated that the participants reported an overall positive user experience of the application, including its ease of use, user confidence and learnability. Most participants reported ease of navigation, particularly when accessing self-help materials and participating in forum discussions. These findings are in line with those that reported a good usability for mobile apps designed to support recovery from EDs (45, 5, 46). However, some minor usability issues were noted, including difficulties with the initial login process and accessing group sessions. These findings are consistent with previous research highlighting the importance of optimizing app functionality to increase user engagement (12, 14). Although people with eating disorders are largely open to using apps to support their treatment, iterative modification and refinement of the app based on user feedback is important to improve its acceptability (14). Qualitative data further supported these quantitative findings and revealed a generally good acceptability among participants. Participants appreciated the structured and interactive nature of the INTERconNEcT-EDs program, particularly the availability of video materials and peer-led forums. An important aspect of the usability evaluation was the ability of the aChiral Content mobile app to foster a sense of support and engagement, a crucial element in digital interventions for EDs (25). However, some participants highlighted challenges with certain technical features, such as the inability to annotate the workbook directly within the mobile app. Future iterations should prioritize optimizing these features to increase user satisfaction and engagement.

The INTERconNEcT-EDs program was designed to provide targeted self-help materials tailored to the needs of individuals at different stages of ED recovery. Participants' responses indicated that the content was perceived as useful, clear and effective in supporting their recovery process. The self-help materials, particularly the workbook and video clips, were highly valued, with many participants reporting increased self-awareness and motivation to engage in behavior change. In addition, the involvement of people with lived experience in the development and delivery of the materials was particularly well received. This approach is consistent with evidence suggesting that peer-led interventions can increase engagement and relatability in mental health interventions (18). Participants noted that hearing from people who had successfully recovered from an ED was inspiring and contributed to a sense of validation and understanding. Recent research highlights that mobile applications for EDs are increasingly integrating peer support and cognitive-behavioral therapy (CBT)-based strategies to improve adherence and engagement (1, 7). The INTERconNEcT-EDs program appeared to be in line with this trend, providing structured and interactive content that supports the therapeutic process while maintaining accessibility.

A key strength of the INTERconNEcT-EDs program was its ability to facilitate social interaction and peer support through interactional guidance. Participants highlighted the value of both the forum group and the videoconference group session, emphasizing the sense of community and shared experience. Previous studies on usability showed that establishing connections with others in recovery from EDs is of significant value. This may be facilitated by incorporating a social networking component into the application, as previously outlined by qualitative studies (14). In the present study, participants reported that peer-to-peer social interactions in the forum can improve personal involvement in recovery. This is consistent with previous research showing that peer support can be a crucial component of ED interventions, helping to reduce feelings of isolation and increase motivation for recovery (21). Previous qualitative usability studies suggested that the degree of interactivity is crucial for online engagement (47) and our findings support the use of peer-led forum groups as tools for facilitating a sense of connectedness between the user and the mobile program. However, while the forum group was widely valued, some participants found the videoconference session challenging due to technological limitations and discomfort with face-to-face interactions in a virtual format. These findings suggest that while synchronous communication methods can be beneficial, providing alternative asynchronous options (e.g., structured discussion boards) may increase accessibility and participation (23). Studies of other ED-related apps, such as Recovery Record and Noom Monitor, have also found that structured peer engagement can significantly improve user retention and perceived effectiveness (48).

A key strength of this study is that it represents the first usability test of the Achiral mobile application for eating disorders (25), specifically designed to provide interpersonal support through online forum groups and interpersonal groups. In contrast to the majority of applications for ED, the Achiral app is designed to be utilized for a range of eating disorders. Additionally, the development of this novel self-help app was approached from the perspective of user-centred design, through a collaborative effort between clinicians, researchers and individuals with lived experiences. Moreover, the involvement of those most impacted by the proposed intervention will contribute to its scalability (25). However, this study has some limitations. First, the sample size was relatively small and non-representative, which limits the generalizability of the findings. While the participants' sample was adequate for the qualitative analysis, future studies should aim to include a larger and more diverse sample to increase external validity and integrate qualitative and quantitative self-report data more comprehensively. Second, the usability study was conducted over a short period of time (four days of app use), which may not fully capture the dynamics of participants' long-term engagement and adherence. Longitudinal research is needed to assess sustained app use and its impact on ED symptoms. Third, the adoption of a deductive thematic framework can limit researcher's ability to explore unexpected or new aspects of the data, with the risk of ignoring themes that do not align with their pre-existing codes (41, 42). Nevertheless, the incorporation of data from semi-structured interviews on participants' usability, in conjunction with the thinking aloud approach, facilitated the richness, transparency and rigor of the qualitative approach. It is also worth noting that although the mobile app was generally well received, some participants experienced technical difficulties, suggesting the need for ongoing usability testing and iterative improvements. Future research should also explore the integration of additional features, such as personalized feedback mechanisms and AI-driven support tools, to further enhance the effectiveness of digital interventions for EDs. In addition, investigating the impact of the self-help app on clinical outcomes, such as changes in ED symptomatology and psychological distress, will be crucial in determining its usefulness in promoting patient change.

## 5 Conclusions

Overall, the usability testing of the INTERconNEcT-EDs program delivered via aChiral Content mobile app, suggests that it is a promising digital intervention for people with EDs. The mobile app was well received, with positive feedback on its content and social support features. By using digital tools and incorporating lived experience perspectives, interventions such as INTERconNEcT-EDs have the potential to bridge gaps in ED care and support individuals throughout their recovery journey.

## Data Availability

The raw data supporting the conclusions of this article will be made available by the authors, without undue reservation.
